# Predictors, barriers, and facilitators to refugee women’s employment and economic inclusion: A mixed methods systematic review

**DOI:** 10.1371/journal.pone.0305463

**Published:** 2024-07-17

**Authors:** Areej Al-Hamad, Yasin M. Yasin, Kateryna Metersky

**Affiliations:** 1 Daphne Cockwell School of Nursing, Toronto Metropolitan University, Toronto, Ontario, Canada; 2 Department of Nursing and Midwifery, College of Health Sciences, University of Doha for Science and Technology, Doha, Qatar; University of Greenwich, UNITED KINGDOM

## Abstract

Refugee women’s employment and economic inclusion have emerged as significant areas of focus, with these women facing unique challenges due to their gender, refugee status, and sociocultural identities. Policymakers and researchers worldwide are giving this issue increased attention. This systematic review uses a mixed methods approach and includes 31 studies to explore the predictors, barriers, and facilitators of refugee women’s employment. The results reveal a pooled employment rate of 31.1% among refugee women. It identifies demographic features, language proficiency, education, and family structure as critical determinants of employment. The qualitative synthesis uncovers three key themes: the meaning and significance of employment; barriers to employment; and facilitators and coping for employment. This study underscores the multifaceted influences on refugee women’s employment. The findings can inform the creation of more targeted interventions, policies, and practices to support refugee women’s employment and economic integration.

## Introduction

Over the last decade, countries worldwide have received significantly increased numbers of refugees [1]. A refugee is a person “who owing to a well-founded fear of being persecuted for reasons of race, religion, nationality, membership of a particular social group or political opinion, is outside the country of his/her nationality and is unable, or owing to such fear, is unwilling to avail himself/herself of the protection of that country [2] p 14. Women with a refugee background are particularly vulnerable, even more than other migrant women and refugee men, because of their gender and refugee status, and social, cultural, political, and personal identities [3, 4]. Policymakers are increasingly focusing on their employment and economic integration. Most agree that their employment is critical for labor market outcomes [5] to offer these women the same privileges, opportunities, rights, and freedoms similar to the hosting countries residences.

Women with a refugee background struggle to find employment opportunities or access entrepreneurial resources in their new host country because of their legal status, refugee backgrounds, and gendered constraints [6, 7]. Additional barriers to employment include language and unfamiliar institutional norms [8]. Refugees frequently arrive in the host country with no valuables or documentation and a lack of work experience in the host country [3, 6, 7, 9]. Because of the financial pressures they face, some refugee women are forced to return to their home country to access funds, capital, or labor and often relying on their social networks back home for support [9]. Additionally, refugees often find employment that does not match their prior skills and experience, resulting in lower occupational status and contributing to poorer health and wellbeing outcomes, particularly among refugee women [10].

Economic inclusion involves expanding economic opportunities to groups that have historically been marginalized or overlooked [11]. Its goal is to enhance the participation of disadvantaged individuals in broader market and business activities, thereby alleviating poverty and elevating their socio-economic standing [12]. This concept is founded on the fundamental belief that everyone, irrespective of their gender, origin, age, social class, or other conditions, should have equitable and complete access to labor and financial markets, as well as economic opportunities [13].

The stigma of the refugee label, compounded by the burden of childcare responsibilities, means that refugee women are more likely than refugee men to struggle to find work [6]. Refugee women’s values, beliefs, and social and personal identities continue to shape processes of marginalization in their new host country, a fact that has intensified discussions about power dynamics [3, 14]. The masculine-driven culture in many remote rural areas and host countries’ gender-equality culture may limit refugee women’s space for agency, establishment, and sense of community [15]. Regardless of migration reasons or the institutional contexts, women with a refugee background tend to enter the low-paying care sector [3].

All of these obstacles can increase refugee women’s marginalization and exclusion, and negatively affect employment opportunities available to them in their new host country. Critical research gaps remain regarding the potential of employment and economic inclusion to empower women with a refugee background socially, economically, and politically [7]. Despite the fact that employment is recognized as essential to integration [9], the limited options and insecure job conditions that women with a refugee background face highlight the harsh realities of their employment experiences [3]. Given that women with a refugee background differ from the immigrant population as a result of their legal status, refugee background, and stigmatized social status, it is important to explore their experiences of employment and economic inclusion. Restrictions in the labor market decrease employment opportunities and earnings for refugees compared to immigrants with more rigorous regulations result in substantial economic hardships for this group [16]. For example, Germany’s asylum system have linked refugees’ residency to their job market participation [17]. Newcomers, especially "tolerated" rejected asylum seekers, rely on ethnic networks for employment, shifting the diversity management burden onto these groups [17]. This masks the exclusionary effects of neoliberal policies, creating an illusion of inclusion by portraying economic interactions as confined within migrant communities [17].

No previous or ongoing systematic review could be located that has investigated the predictors, barriers, and facilitators of refugee women’s employment and economic inclusion in their new host country. A comprehensive knowledge synthesis is vital to identify areas of disparity along with known barriers and facilitators, with the goal of guiding future practice interventions and research. This mixed methods systematic review (MMSR) fills important gaps in the literature and is the first to synthesize recently published evidence relevant to the predictors, barriers, and facilitators for refugee women’s employment and economic inclusion while also taking into account their experiences and perspectives. The research questions guiding this review included:

1. What are the rates and predictors of refugee women’s employment and economic inclusion in their new host country? (Quantitative question)
2. How do women with a refugee background describe their experiences of employment and economic inclusion, employment barriers and facilitators, and the impact on their health and well-being in their new host country? (Qualitative question)
3. What can be inferred from the qualitative synthesis to explain predictors, barriers, and facilitators that affect refugee women’s employment, economic inclusion, and the impact on their health and well-being in their new host country? (Mixed methods question)

## Materials and methods

Sandelowski’s segregated approach from the Joanna Briggs Institute’s (JBI) Mixed Methods Systematic Reviews (MMSR) was used for this review [18]. The approach recognizes the various ontological and epistemological presuppositions that underlie qualitative and quantitative research [18]. An MMSR synthesizing quantitative and qualitative data can establish convergent validation (or triangulation) between qualitative and quantitative studies in a common area of research [18]. The segregated approach to mixed method synthesis consists of separate syntheses (quantitative and qualitative synthesis) of each component method of the review [19]. Combining and/or comparing a review’s resultant qualitative and quantitative findings can provide unique, innovative, and comprehensive insights into the findings [19]. This segregated approach to our MMSR has the potential to provide unique insights and actionable findings that will inform policy and practice by providing evidence for decision makers [19]. Sandelowski argues that recognizing the differences of various ontological and epistemological presuppositions is crucial for aligning research methodologies with objectives, leading to more coherent and insightful investigations that honor the complexity of human experiences [18, 19].

### Search strategy for identifying studies

With the assistance of an academic librarian, we conducted a preliminary search of MEDLINE, PROSPERO, the Cochrane Database of Systematic Reviews, and the JBI Database of Systematic Reviews and Implementation Reports for any relevant systematic reviews and found no reviews examining the predictors, facilitators, and barriers to refugee women’s employment, economic inclusion, and the impact of their social, cultural, political, and/or personal identities on their experiences. A priori protocol has been published in PROSPERO (Registration ID: CRD42023413388).

We executed a comprehensive and systematic search strategy using the Preferred Reporting Items for Systematic Reviews and Meta-Analyses guidelines [20] and the Joanna Briggs Institute methodology for mixed methods systematic reviews [21]. We searched the following databases: (1) Academic Source Complete, (2) Business Source Elite, (3) Business Source Complete, (4) Family and Society Studies Worldwide, (5) PsycINFO, and (6) Scopus. Boolean operators AND/OR were used to narrow or broaden the search using a combination of the keywords. Search terms included: (*refugees* or *asylum seekers*) AND (*women* or *female* or *woman* or *females*) AND (*employment* or *jobs* or *work* or *career*). The following limiters were used: publications in the English language, peer reviewed, and the date of publication between January 1^st^ 2011 and March 31, 2023. We also manually screened the reference lists of included studies for other relevant studies for inclusion. All search results were collated and uploaded into the bibliographic software, Endnote (V.20), and duplicates were removed before data extraction.

### Inclusion and exclusion criteria

We limited the review to empirical studies in peer-reviewed journals and grey literature published in English from January 1^st^ 2011 to March 31, 2023 to capture the recent refugee waves resulting from invasions, wars, and international conflicts including the Middle East, the Russian invasion of Ukraine, and Southeast Asia. We utilized the PICO mnemonic (population, intervention, comparator, outcomes) for the quantitative component and the PICo mnemonic (population, phenomenon of interest, context) for the qualitative component. Participants in the quantitative and mixed-methods component were women with a refugee background (18 years old or older). Interventions were considered if they involved any vocational training program or employment models. Both active and passive comparison groups were included as comparator(s). Case reports, study protocols, discussion papers, editorials, and literature reviews, as well as any studies with a target population that were not refugee women, written in a language other than English, or published before 2011 were excluded.

### Study selection and quality assessment

This review considered quantitative, qualitative, and mixed methods studies. Two team members (AA and KM) independently screened titles and abstracts according to eligibility criteria. Next, the same two members assessed in detail full-text articles of all eligible studies against the eligibility criteria. The yields from the search results are presented in a PRISMA flow diagram with a clarification of various stages of article screening to reach the final included studies [20], including reasons for exclusion. The JBI Critical Appraisal Checklist was used to appraise the selected articles with two reviewers independently appraising the included studies (AA and YY) using the Joanna Briggs Institute critical appraisal checklist for analytical cross-sectional studies [22] and qualitative research [23]. (See S2 File for the critical appraisal results). Any discrepancies or disagreements that arose during the review were settled through conversation or with the help of a third reviewer (KM) to confirm analyses and minimize biases and preconceptions.

### Data extraction and data synthesis

Data were extracted using the JBI standardized quantitative and qualitative data extraction tool. Data were retrieved from studies and contained specific information about the populations, study procedures and methods, and results that were relevant to the review questions and aims. A research assistant and one reviewer (AA) carried out data extraction, and a second reviewer verified the extraction (YY). See the S1 File for the extraction table of the included studies The quantitative and qualitative extracted data were first analyzed and synthesized separately before integrated findings were prepared using a matrix approach for the comparison and to evaluate their complementarity [18]. The research team together with the two research assistants continually verified and validated the data extraction tools to ensure their completeness and clarity based on each team member’s experience or expertise in quantitative or qualitative research design.

### Quantitative synthesis

This synthesis includes studies exploring factors affecting refugee women’s employment and economic inclusion, including barriers and facilitators. The standardized data extraction tool for prevalence and incidence available in JBI SUMARI was used to conduct the meta-analysis module, and the standard *I*2 tests were used to assess heterogeneity. The results of the meta-analysis were described using narrative.

### Qualitative synthesis

Two reviewers (AA and KM) conducted the qualitative synthesis using a meta-aggregative approach using Joanna Briggs Institute System for the Unified Management, Assessment and Review of Information using JBI SUMARI [18, 24–27]. This process involved three steps: coding text and findings from eligible studies; developing categories based on similarities and constant comparison; and then combining the categories to produce synthesized findings. The Confidence in the Evidence from Reviews of Qualitative research (CERQual) approach was used to guide the overall assessment of the qualitative synthesis and to ensure confidence in the findings for informing decisions about guideline development and policy recommendations [28].

### Meta-integration

After the separate quantitative and qualitative syntheses, we performed a meta-integration to juxtapose synthesized quantitative results with the qualitative synthesized findings [29]. No data transformation was conducted and we aimed to aggregate the studies so that statistical inference can be strengthened by qualitative synthesis and also aimed to provide meaningful and practical guidelines for practice and policymaking [29]. A matrix approach was adopted and revealed complex connections between quantitative and qualitative data [29]. The matrix strategy allows us to map the review findings and perform a side-by-side comparison to identify similarities and differences that were depicted in both quantitative and qualitative findings. If data from quantitative and qualitative synthesis relate to each other, we classify these as “complementary.” Otherwise, we classify the data as “conflicting” and requiring more investigation [29]. All authors contributed to the data synthesis and integration.

### Methodological quality

Two reviewers (AA and YY) separately carried out quality assessments on the included studies, making use of the JBI critical evaluation checklists. These checklists had eight questions for analytical cross-sectional studies, 11 questions for cohort studies [22], and 10 questions for qualitative studies [23]. In cases where the reviewers did not agree, a third reviewer was brought in (KM), and then consensus was reached through discussion. If an answer was given as (not applicable) or (unclear), it was interpreted as the criteria not being fulfilled. Studies that garnered a score below 60 percent based on the quality assessment queries were not included. One mixed-method study warranted an evaluation using both cross-sectional and qualitative appraisal tools as a result of the study type. The methodological quality scores in detail, the questions, and the corresponding answer key for each study according to its design are provided in the *S2 File for the critical appraisal of the included studies*. The scores for the methodological quality for the 19 qualitative studies ranged from 70 to 90 percent and for the 11 quantitative studies ranged from 63.6 to 100 percent and 77 percent for the mixed method study.

## Results

**Fig 1:**
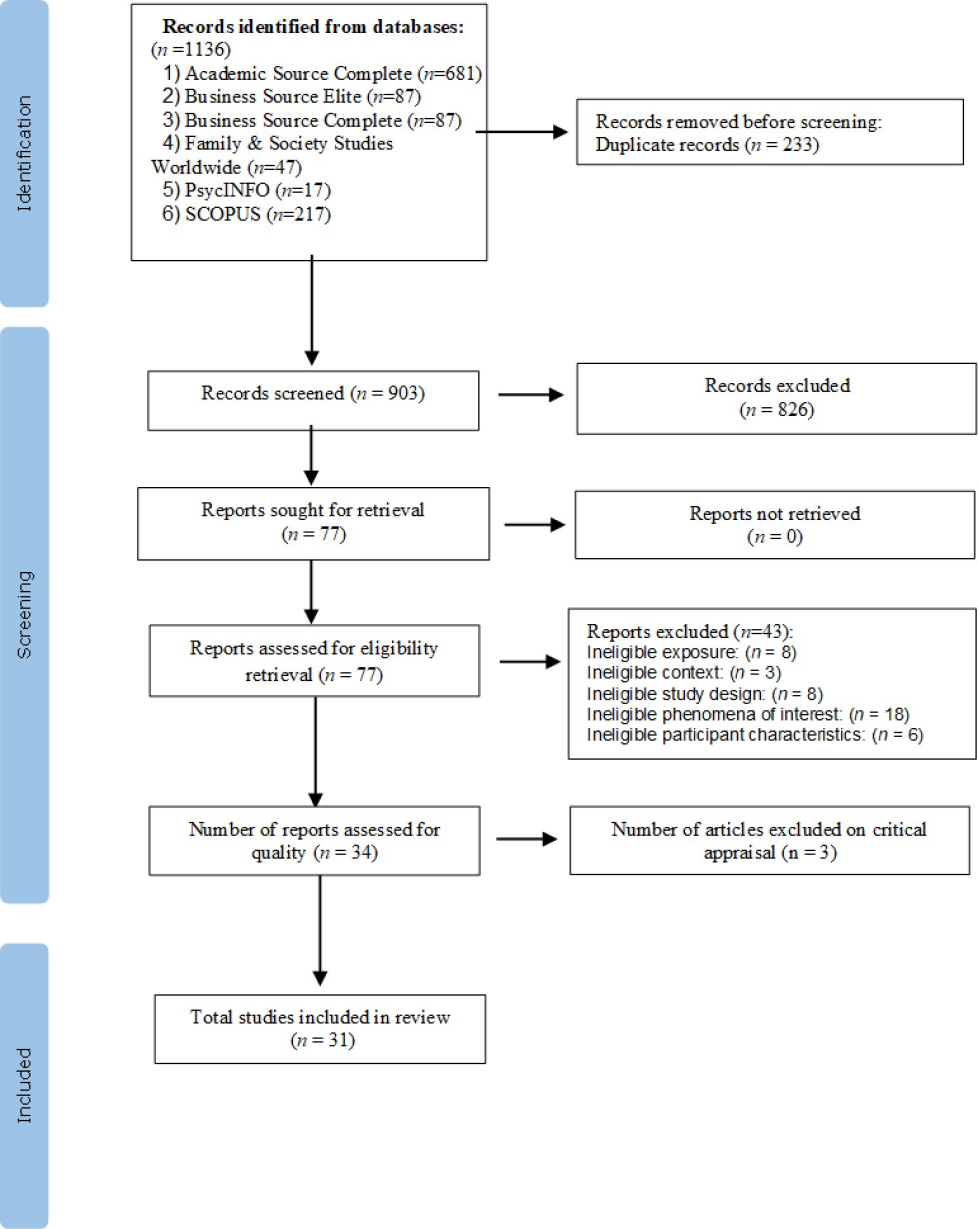
PRISMA flow diagram of the study selection process.

The search yielded 1,136 citations, 233 of which were duplicates. The remaining 903 records were screened for relevance based on title and abstract, and 34 reports were retrieved for full-text review. We also examined the reference lists from these reports but found no additional papers. Following the critical appraisal, three additional studies were excluded because their scores were less than 60 percent based on the quality assessment queries. A total of 31 papers qualified for inclusion in the final review. See Fig 1 for the PRISMA chart.

### Characteristics of included studies

This review involved 31 studies which include 11 quantitative, 19 qualitative, and one mixed-methods study. Of the included studies, the highest number of studies were conducted in the USA [8, 30–33], and one that involved both the US and the Netherlands [34]. Four studies were conducted in Sweden [15, 35–37], including one that spanned both Lebanon and Sweden [38]. Australia also had four studies [39–42]. Jordan was represented in three studies [43–45] and jointly with Lebanon in one study [46]. Two studies were conducted in Canada [47, 48] and South Africa [49, 50] respectively. Two studies involved multiple countries, including the Democratic Republic of Congo, Burundi, and Zimbabwe [51] and the Eurozone of the European Union [52]. One study was conducted in each of the following countries: Austria [53], Denmark [54], Ireland [55], South Korea [56], Turkey [57], and the UK [58].

The quantitative studies mainly employed a cross-sectional survey design (*n* = 6), and retrospective and prospective cohort designs (*n* = 5), though one study combined a cross-sectional survey with a phenomenological approach in a mixed-methods study. These studies covered an extensive sample of 29,255 women with a refugee background, with individual study samples ranging from 159 to 6,227 participants. The qualitative studies predominantly used descriptive designs (*n* = 10), incorporating in-depth interviews or a blend of interviews and focus groups. They engaged specific qualitative approaches such as feminist grounded theory (*n* = 1), narrative inquiry (*n* = 2), ethnography (*n* = 2), case study (*n* = 1), community-based participatory research (*n* = 1), phenomenology (*n* = 1), and hermeneutic research (*n* = 1) to delve into particular aspects of refugee women’s experiences. See S3 File for the PRISMA checklist.

#### Quantitative findings

When we focus on the employment rate of women with a refugee background as documented in 11 quantitative studies and the quantitative arm of one mixed-method study, we find a multifaceted landscape where employment rates fluctuate significantly according to geographic location and demographic characteristics. Through an analytical lens, we see the complexity of refugee women’s employment by dissecting various contributing factors such as demographic and well-being factors, societal and cultural factors, and the availability of governmental and nongovernmental resources.

### Refugee women employment rate

**Fig 2:**
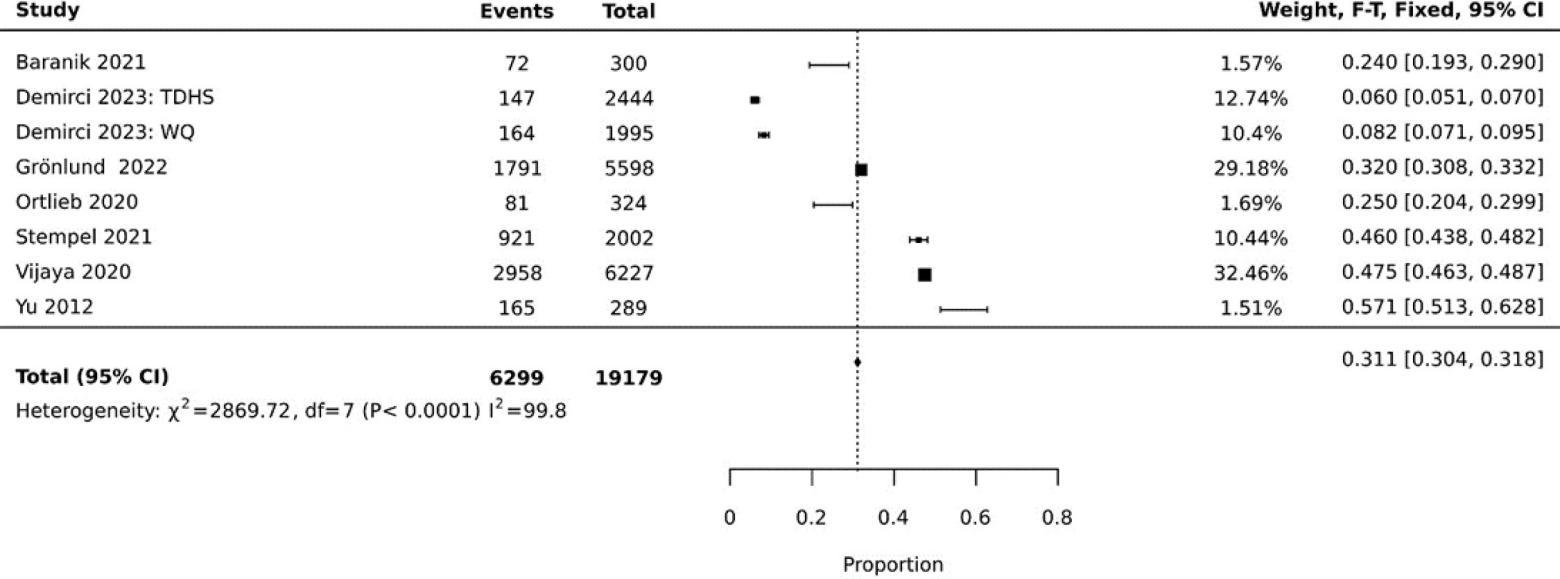
Refugee women employment forest PlotDemographic and well-being factors.

Out of the 12 studies incorporated in the quantitative synthesis, seven either explicitly mentioned the employment rate or we deduced the rate. The employment rates for women with a refugee background depicted in these studies showed a substantial variation depending on the countries and demographics under study. Table 1 summarizes the refugee women’s employment rate based on the host country in the included studies. When pooling data from these seven studies (with one study contributing two separate data sources), we computed an average employment rate of 31.1 percent, with a 95 percent confidence interval ranging from 30.4 to 31.8 percent (refer to Fig 2 for details).

**Table 1:** Employment rate per country.

Country	Employment Rate
Turkey	6% - 8.2%
Lebanon and Jordan	24%
Australia	24.9%
Sweden	32%
USA	46% - 47.5%
South Korea	57%
**Pooled Data**	**31.1% (95% CI: 30.4% - 31.8%)**

Several demographic factors were associated with employment such as age, health perception, household size, dependent children, and education. For instance, six studies found that older age is a constraint to employment [8, 30, 32, 41, 57]. One study reported that child-bearing age and parenthood at age 30 have a significant negative impact on employment [35]. Furthermore, mental and physical health were both significant predictors for employment opportunities [33, 58]. For example, Government Assistance Refugee (GAR) were less likely to have opportunities to demonstrate their skills in the workforce, which could have a negative impact on their mental health [48]. Additionally, the skill development programme to foster employment has a considerable positive impact on the participants’ health and personal growth [45]. The structure and characteristics of a family also wield substantial influence on the employment status of women with a refugee background. For instance, marital status was found to be a facilitator of employment among refugee women in Sweden because marital status can provide additional social and economic support, easing integration into the job market [32, 35]. Conversely, having a larger household size was identified as an impediment to employment [57], while having more children posed significant barriers to employment [41]. Cheung and Phillimore [58] reported that in the UK, women with a refugee background with dependent children were less likely to be employed, a fact that was echoed by studies in the United States [8], and South Korea [56], where researchers found that having young children adversely affected refugee women’s employment prospects.

Education was a crucial determinant in refugee women’s employment. Research conducted across various countries, including Turkey [57], Sweden [35, 36], Australia [41], and the US [8, 33] indicates that higher levels of education are positively correlated with improved employment prospects for women with a refugee background. However, despite possessing advanced educational qualifications, refugee women often encounter challenges in capitalizing on their education to secure economic opportunities. Stempel and Alemi [33] highlighted that such difficulties in converting educational attainments into economic capital could pose constraints on the employment opportunities available to women with a refugee background. The employment of refugee women has profound implications for their overall wellbeing, including their mental health [42, 45, 48, 58]. Securing meaningful and appropriate employment not only facilitates economic stability but also enhances self-esteem and social integration, which are crucial for mental health [48]. However, when refugee women find themselves in jobs that do not match their qualifications or previous experience, it can lead to underemployment, which often results in stress, anxiety, and a diminished sense of self-worth [48]. The constant challenge of navigating a new cultural environment and facing workplace discrimination can exacerbate feelings of isolation and depression [48].

### Societal and cultural factors

Several societal and cultural factors played pivotal roles in affecting refugee women’s employment prospects. The region of origin and cultural background of refugees were significant determinants that can influence their integration into host countries and their ability to secure employment [33, 35, 41]. Additionally, the geographical location within the host country itself can offer varying employment opportunities to women with a refugee background, as studies conducted in Turkey [57], Australia [41], and the United States [30, 32] found. Refugee women’s employment experiences were significantly shaped by their region of origin and cultural background. In developed areas like Europe, North America, and Australia, they faced challenges such as cultural and language barriers, unrecognized qualifications, and restrictive gender norms, all of which can limit their job opportunities. In other regions, less stringent regulations may exist, but social integration and acceptance still present substantial hurdles. Furthermore, the employment prospects of refugee women varied based on cultural background; those from conservative cultures often struggle more due to strict gender norms, while women from cultures with higher female labor participation may find it easier to adapt to work environments.

Work experience in the host country [41] and the duration of stay were also significant factors, with studies demonstrating that these attributes enhanced the employment prospects of women with refugee background [8, 30, 33, 35]. In contrast, host countries’ societal attitudes regarding refugee women’s rights and roles served as obstacles to their employment [46].

Language proficiency was another critical issue. Proficiency in the host country’s language has been found to facilitate employment among refugee women [8, 32, 33, 57, 58]. Building a network of personal contacts, especially with locals in the host country, has also been shown to positively affect employment opportunities for refugee women [56].

### Availability of government and nongovernmental resources

The effectiveness of governmental support stands as a significant predictor for employment among women with a refugee background. Extended case management was found to facilitate employment among refugee women in the US [32]. Extended case management, spanning two years, can significantly enhance refugees’ employment prospects. This comprehensive support model provides ongoing guidance, which helps refugees navigate complex job markets, overcome language barriers, and acquire necessary local qualifications [32]. Consistent assistance over two years allows for sustained personal development and skill acquisition, fostering a better alignment between refugees’ capabilities and job opportunities [32]. While public employment agencies bolster the probability of refugee women securing employment, receiving governmental benefits tends to diminish the vigor of the women’s job search activities [56]. For example, Social Security Benefits for north Korean female refugees in South Korea have a negative impact on wages [56]. Refugee women anticipating these benefits tend to avoid pursuing jobs with higher wages to remain eligible for the benefits [56]. This behavior makes it challenging for North Korean refugees to secure employment with wages significantly exceeding the threshold for receiving the benefits [56]. Governmental initiatives can also result in diverse employment outcomes, including work-first policies, which are often influenced by gender [41, 54]. The work-first policy in Denmark that mandates refugees to actively search for jobs and participate in on-the-job training upon arrival has implications that are often influenced by gender due to stigma. This policy assumes that all refugees can immediately enter the workforce, which overlooks the distinct challenges faced by many women [54].

When it comes to nongovernmental resources, an increase in employment has been associated with certain factors including the engagement in mass media and associations. For instance, Demirci and Kırdar [57] found that membership in the host country associations positively impacted employment among women with a refugee background. Additionally, engagement with mass media, such as job announcements in the public domain and internet searches, served as instrumental avenues for employment opportunities for this population [56].

#### Qualitative findings

An authentic synthesis of qualitative findings, consolidating data from primary studies, was performed. This was succeeded by the creation of a meta-aggregative flowchart. This chart visualizes the findings, their categorization, and ultimately, the assembly of these categories into comprehensive, synthesized findings. Through the qualitative synthesis of the qualitative studies, we identified three key synthesized findings, which collectively illuminate the nuanced dynamics that underpin refugee women’s employment experiences. These include: (1) the meaning and significance of employment; (2) barriers to employment; and (3) facilitators and coping for employment.

### Synthesized finding 1: The meaning and significance of employment

The synthesized finding discusses the meaning of employment as a significant factor for refugee women’s health, well-being, and autonomy [42, 45, 55]. Employment fosters dignity, personal growth, and shifts in gender roles [44, 55]. Moreover, it’s seen as an empowering mechanism that helps overcome language barriers and achieve financial independence [38, 43]. Practical achievements such as acquiring a driver’s license have the potential to enhance refugee women’s agency, mobility, work prospects, and community ties [15]. Simultaneously, employment facilitates local interactions, language acquisition, and understanding the host country’s social norms [42]. Huq and Venugopal [40] emphasized the significance of entrepreneurial journeys among women with a refugee background, underscoring their resilience and capacity for self-reconstruction. However, not all findings were positive. Ghorashi [34] noted that the distinct feeling of being “other” and the underappreciation of refugee women’s skills often create emotional barriers to assimilation. Additionally, refugee women’s focus tends to be on immediate survival needs rather than long-term professional advancement [50].

This synthesized finding reveals employment’s critical role in enhancing refugee women’s personal growth, empowerment, autonomy, well-being, and cultural integration. Although it is typically an economic necessity, employment also aids in overcoming language barriers and understanding the host country’s norms. However, feelings of alienation and overlooked skills often impede emotional connection to the host culture and eventually affect refugee women’s interest in seeking employment and economic integration.

### Synthesized finding 2: Barriers to employment

This synthesized finding presents an analysis of the barriers that women with a refugee background face in gaining employment in their host countries. Multiple studies identify language barriers and lack of recognized credentials as key obstacles [30, 40, 47, 48, 51, 55]. Systemic obstacles, such as allocation policies, inefficient resettlement agencies and interpretation services, and lack of overall support, have also been recognized as substantial challenges to employment [30]. Spehar [37] shed light on the devaluation of refugee women’s skills and the psychological barrier of “starting over” as additional obstacles.

Experiences of xenophobia, violence, and issues with document accessibility, sexism, and work-life balance impede refugee women’s employment [50]. Their dependency on public benefits, and their social isolation, family separation, and limited support further compound the problems, restricting their employment opportunities [30]. Kikulwe, Massing [47] noted the impact of the loss of security and autonomy on refugee women’s employment, while Darawsheh, Bewernitz [30] and Smit and Rugunanan [51] brought attention to the host country’s discriminatory practices.

Structural and patriarchal cultural factors also present barriers to refugee women’s employment including community expectations of women to have a caring attitude and to work with children and the elderly [15]. During the COVID-19 pandemic, these challenges were amplified by gender and racism-based arrangements in fragmented and limited labor markets [52]. These arrangements highlight how structural inequities in the labor market rooted in gender and racial discrimination can lead to unequal economic impacts, which were particularly pronounced during the disruptions caused by the COVID-19 pandemic. Finally, domestic violence experiences have also been identified as detrimental to refugee women’s employment prospects [39].

The data provide a perspective on the systemic, cultural, and personal challenges that these women encounter when pursuing employment. Key barriers for refugee women’s employment included: language difficulties, unrecognized qualifications, and systemic inadequacies, like resettlement policies and insufficient support. Experiences of discrimination, violence, and societal expectations further compound these issues. The COVID-19 pandemic added more strain, further marginalizing women with a refugee background in the workforce.

### Synthesized finding 3: Facilitators of employment and coping strategies

The studies for this synthesized finding highlighted the resourcefulness women with a refugee background who adapt to their circumstances and leverage various personal and social assets to secure employment. These resourcefulness initiatives include entrepreneurship initiatives, like opening hair salons or street vending [49] and specific career choices such as teaching [38]. Networking also emerged as a significant employment facilitator [43]. Furthermore, opportunities to develop postmigration human capital, including language proficiency, personal agency factors including personal motivation and goals and professional skill sets, digital literacy, and skills development programs, positively affected employment outcomes [38, 53]. Language, education, and work experience upon arrival also provided flexibility for labor market entry [48]. Access to transportation, such as having a driver’s license, broadened refugee women’s work prospects [15]. There’s an intricate relationship between employment and education, with each aspect reciprocally influencing the other to enhance refugee women’s employment [42].

The data revealed a range of strategies that women with a refugee background employ when they’re faced with employment constraints. These livelihood strategies included engagement with social media, part-time or sporadic work [51], and employment in traditional female roles such as in hospitality, retail, and performing menial tasks [31, 49]. Adaptive cognitive reasoning and behavior modification (e.g. activity scheduling, psycho‐education, problem‐focused and mindfulness) after women experience domestic violence also assisted in enhancing their employment prospects [39]. Engaging in career development, language proficiency, and digital literacy foster refugee women’s social engagement, employment, and economic inclusion to meet individual and family needs [38].

## Discussion

This systematic review and meta-analysis offers insight into the multifaceted circumstances and contributing factors that shape the employment rates of women with a refugee background. The meta-analysis quantitatively appraised the landscape of employment among refugee women through the lens of 11 studies and the quantitative branch of a mixed-method study. The ensuing discussion compares these quantitative results with the qualitative findings derived from a thematic synthesis of the qualitative studies. The comparison reveals distinct consistencies and alignments, highlighting the employment experiences of refugee women.

Our quantitative findings unveiled an average employment rate of 31.1 percent among refugee women, corroborating the view that female foreign nationals face numerous challenges that may obstruct their employment and integration as the qualitative synthesis found [47, 49, 52]. The findings indicate significant variation in the employment rates of women with a refugee background across different countries. Several factors may contribute to these differences, including the host country’s institutional and policy constraints, gender role attitudes, economic conditions, labor market dynamics [59], and the availability of support services for refugees [60].

Several demographic features including age, language proficiency, education, and family structure as well as well-being factors identified from the quantitative analysis as critical determinants of refugee women’s employment—a trend also echoed in the qualitative synthesis. For example, age was a factor impeding employment [30, 57] corroborating the qualitative finding of older refugee women encountering barriers to employment [30]. These barriers and factors are consistent with previous studies that point out the low employment rate and the lack of meaningful economic opportunities for women with a refugee background [5, 61].

Conversely, the qualitative synthesis highlighted the importance of employment for refugee women’s health, well-being, empowerment, and autonomy [38, 42, 45, 55] aligning with the quantitative analysis that revealed good physical and mental health as significant predictors of employment [33, 58]. Therefore, employment that aligns with their skills and experience is vital for supporting the mental of refugee women, as it fosters a sense of purpose, enhances their social networks, and provides a crucial sense of community and belonging. Likewise, the influence of language proficiency and family structure and characteristics on employment opportunities aligns across both quantitative [35, 57] and qualitative studies [30, 47, 48]. These finding are consistent with previous studies on the role of language proficiency and its impact on women’s employability [62–64].

While quantitative studies found education to be positively correlated with refugee women’s employment prospects [33, 57], they also highlighted that despite possessing advanced educational qualifications, women with a refugee background often face difficulties in securing employment [33]. This finding complements the qualitative synthesis, which highlighted unrecognized and undervalued credentials as a significant barrier to refugee women’s employment [47, 51, 55]. These findings are congruent with previous studies that describe barriers stemming from refugees’ precarious legal status and devalued credentials [61, 65–67].

A comparison between the quantitative and qualitative findings reveals noteworthy similarities regarding the barriers to employment for women with a refugee background. The quantitative results highlight the role of societal and cultural factors, including refugee women’s region of origin, cultural background, and host country’s geographical location [33, 35, 41]. These parallel the qualitative findings that elucidate language barriers, lack of recognized credentials, and systemic obstacles as key hurdles [47, 48, 51]. Both sets of findings converge on the idea that structural and patriarchal cultural factors, discriminatory practices, and societal expectations present additional barriers to refugee women’s employment [15, 30, 51]. These structural, contextual, and discriminatory practices echo what Fuller and Martin [68] claimed about the human capital attributes and household context that shape immigrant employment trajectories.

The societal and cultural factors identified in the quantitative studies aligned with those explored in the qualitative synthesis. Notably, the language proficiency in the host country was found to facilitate employment in both quantitative [57, 58] and qualitative studies [38, 40, 51]. Personal contacts in the host country, such as networks, were also shown to have a positive impact on employment opportunities in both the quantitative [56] and qualitative findings [43]. This finding is consistent with previous studies that concluded social contacts, in particular contacts with host-country natives, are important for immigrant employment and increase the likelihood of male and female employment [69].

In terms of facilitators and coping for employment, the quantitative findings suggest that support from governmental and nongovernmental resources, work experience in the host country, language proficiency, and networking impact the employment prospects of refugee women positively [30, 32, 56]. These align with the qualitative findings, which illuminate the resourcefulness of refugee women in their quest for employment as they leverage entrepreneurship, network, engage in career development program, and develop postmigration human capital [38, 53]. These findings are consistent with other studies that underline the resilience of women with a refugee background, demonstrating their ability to navigate a complex socio-cultural landscape and overcome work-related challenges influencing their health and employment [70, 71].

Concerning governmental and nongovernmental resources, the quantitative analysis recognized the effectiveness of government support as a significant predictor for refugee women’s employment [32]. Conversely, the qualitative synthesis emphasized systemic obstacles, like inefficient resettlement agencies, as substantial challenges to employment [30], highlighting the need for an improved understanding of the relationship between refugee women and the public sector. These findings are congruent with previous studies that highlight how working with the nongovernmental organizations (NGOs), as well as personal and social ties, promote refugee women’s employment and strengthen their resilience by acquiring personal assets [5, 72].

Finally, there is a notable coherence between the quantitative and qualitative findings, underscoring the multifaceted influences on refugee women’s employment. Refugee women’s employment is influenced by various predictors, barriers, and facilitators. These findings portray a complex picture of the employment experiences of refugees, underlining the challenges, coping mechanisms, and empowering aspects of work. Our comprehensive review of the literature on refugee employment reveals a significant gap concerning the specific needs of women with a refugee background. While existing research underscores the challenges faced by this group, there is a pressing need for more nuanced and targeted interventions that address these unique obstacles. Particularly, the role of personal agency factors such as motivation to work and readiness to engage in entrepreneurship has been largely overlooked. Understanding how these internal dynamics interact with external barriers is crucial for developing effective strategies that support refugee women in securing employment or starting businesses in host countries. This insight could lead to more tailored programs that not only facilitate job placement but also empower these women to achieve long-term economic independence and success.

### Review implications

The findings of this systematic review and meta-analysis have critical implications across the domains of policy and administration, practice, and research. From a policy and administration perspective, the evidence underscores the need for responsive and targeted policies that address the specific barriers that women with a refugee background encounter in their journey toward employment. These might include gender-sensitive and inclusive policies that facilitate the recognition and validation of foreign qualifications, language acquisition, and the development of skills relevant to the host country’s job market. Administrative procedures need to be mindful of the intersectional issues that refugee women face, which necessitate cross-sectoral collaborations to provide comprehensive support. Host governments must enact and enforce policies that combat xenophobia, discrimination, and violence against women with a refugee background, ensuring their safety and protection in the workplace. They also should raise awareness and provide training to employers, communities, and service providers on gender equality, cultural sensitivity, and the rights and contributions of refugee women.

In terms of practice, the findings highlight the crucial role of health and social practitioners working with refugee women to understand the barriers and facilitators to their employment. Settlement agencies and organizations working with refugees may need to take proactive steps to help women navigate these complexities, whether that involves arranging language classes, providing resources for credential recognition, or offering career advice and guidance. Developing language training programs specifically tailored to the needs of women with a refugee background, and focusing on improving their language proficiency to facilitate employment opportunities will also be crucial. We also need to establish streamlined processes for recognizing and validating the educational credentials of refugee women, ensuring that their qualifications are acknowledged and utilized in the host country’s job market.

Finally, in terms of research, the findings highlight the need for further exploration of the intricate dynamics influencing refugee women’s employment rates. Future studies should aim to unpack the mechanisms underlying the factors we’ve identified, examine the efficacy of different interventions and employment models, and prioritize comprehensive designs that encompass diverse geographic locations and study populations. Longitudinal studies might offer valuable insights into the long-term trajectories of refugee women’s employment and the factors influencing their progress. Ultimately, continued research in this area is vital for informing effective, evidence-based policies and practices to improve the employment prospects of women with a refugee background.

### Review limitation

While our mixed-method systematic review, based on Sandelowski’s (2006) segregated approach, provides unique insights into refugee women’s employment, it also has inherent limitations. The use of only English-language studies and studies published from 2011 onward could have excluded potentially relevant research. In particular, valuable insights might be overlooked from non-English-speaking countries where the refugee situation is significant. Our review may also be impacted by publication bias, where studies showing significant results are more likely to be published and therefore included in our review. Finally, meta-integration, while a strength in revealing complex connections, still depends on the quality and relevance of both quantitative and qualitative syntheses.

## Conclusion

This mixed-method systematic review and meta-analysis offers a comprehensive perspective on the barriers, facilitators, and predictors of refugee women’s employment and economic inclusion. This review sheds new light on the unique challenges women with a refugee background face when it comes to employment and economic inclusion. Also, the study offers a better understanding of the factors that affect refugee women’s employment and economic inclusion to inform future employment models, policy, and practice implementation. The review findings draw inferences about the programs/models, design, features, and implementation contexts that are inclusive, gender specific, and conducive to offer effective refugee women’s employment and entrepreneurship programs/models. The results point toward potential best practice guidelines for employment organizations working with refugee populations. The findings can be translated into a set of practices, policy recommendations, and theoretical frameworks that can lead to better outcomes for refugee women’s employment and economic inclusion and to promote their identities and opportunities in their new host country. By continuing to refine our understanding of the complex factors shaping refugee women’s employment experiences, we can contribute to the creation of more inclusive, equitable societies.

## Supporting information

S1 File Characteristics of included studies. (DOCX)

S2 File Critical appraisal of the included studies. (DOCX)

S3 File PRISMA 2020 checklist. (DOCX)
