# Supplementary material for: Predictors, barriers, and facilitators to refugee women’s employment and economic inclusion: A mixed methods systematic review
Source: PLoS One. 2024 Jul 17;19(7):e0305463. doi: 10.1371/journal.pone.0305463 (PMC11253926; doi:10.1371/journal.pone.0305463)
Supplement: S1 File — (DOCX) [file pone.0305463.s001.docx]

**Predictors, Barriers, and Facilitators to Refugee Women’s Employment and Economic Inclusion: A Mixed Methods Systematic Review**

***Supplementary File 1***

**Characteristics of Included Studies**

| **Study** | **Population & Sample size** | **Country** | **Research design** | **Data collection** | **Main description of employment results**  **(Refugee women)** |
| --- | --- | --- | --- | --- | --- |
| Almakhamreh S, Asfour HZ, Hutchinson A. 2022. | Syrian refugee women (n=15)  Social workers (n=6) | Jordan | Qualitative descriptive | In-depth interviews and focus groups with refugee women and social workers. | 1. Meaning and significance of employment: Refugee women reported feeling more empowered and independent financially than they would have in their home country. Refugee women benefited from patriarchal gender roles by feeling more empowered. 2. Barriers to employment: NA 3. Facilitators and coping strategies: Gender was used as a means of securing, Syrian refugee women connected with the host country in a collective manner. employment, particularly when done through networking. |
| Arendt JN. 2022. | Syrian and Eritrean refugees  (*n* _Total_ =9,250)  (*n _R_*_W_ = 4,765) | Denmark | Retrospective cohort | - Governmental data records - Nine months before and after October 2016 | 1. Employment rate: NA 2. Predictors: work-first policy has limited impact on women employment while it has a significant impact on men employment |
| Baranik LE. 2021. | Native-born  (*n* _Total_ =2,400)  Syrian refugees (*n* _Total_ = 600)  (*n _R_*_W_ = 300) | Lebanon and Jordan | Cross- sectional survey | - Nationally representative data from the Arab Barometer - From 2016–2017 | 1. Employment rate: 24% 2. Predictors: Attitudes towards women's rights and roles |
| Bradley L, Bahous R, Albasha A. 2022. | n = 20 Syrian refugee women  (Lebanon - n=10 and Sweden - n=10) | Lebanon and Sweden | Qualitative descriptive design | - Semi-structured Interviews | 1. Meaning and significance of employment: Engaging in career development is empowering and plays a role in overcoming language barrier. Teaching as a career choice. 2. Barriers to employment: Language barrier 3. Facilitators and coping strategies: Language, digital literacy and professional experiences are important skills for economic inclusion and employment. |
| Carlbaum S. 2022. | n = 36 (migrant women. local staff, educators) | Sweden | Ethnographic research | - Semi-structured Interviews | 1. Meaning and significance of employment: a sense of agency, independence and a sense of community. 2. Barriers to employment: structural and patriarchal cultural factors including caring attitude, working with children and the elderly. Refugee Women struggle to integrate, take longer to learn Swedish. Due to the concept of "otherness" in rural areas, a lack of work opportunities, and social isolation, many refugee women leave these areas. 3. Facilitators and coping strategies: Having a driver's licence gives refugee women a sense of agency, opens up work prospects (especially for masculine jobs), and a sense of independence and a sense of community. |
| Caspersz D, Casado R, Kaplanian C, Fozdar F, Baldassar L. 2022. | N=9, Clinical Psychologist group n=4  Social worker group n = 5 in Australia | Australia | Hermeneutic research | - In-depth interviews and observations | 1. Meaning and significance of employment: The meaning of domestic violence experience and its impact on employment prospect. 2. Barriers to employment: NA 3. Facilitators and coping strategies: Interpersonal and intrapersonal processes to seek employment after domestic violence, adjusting cognitive reasoning and behaviours to enhancing employment prospect. |
| Cheung SY, Phillimore J. 2017. | No specified ethnicity refugees (*n* _Total_ = 5,742) | UK | Retrospective cohort | - Survey of New Refugees - From 2005-2007 | 1. Employment rate: NA 2. Predictors: Women refugees with dependent children, language proficiency and good health |
| Ćatibušić, B, Gallagher F, Karazi S. 2021. | n = 26 Syrian refugee in Ireland | Ireland | Qualitative descriptive design | - Semi-structured Interviews | 1. Meaning and significance of employment: language for employment, social interaction, and access to accurate information, sense of personal well-being, dignity and autonomy. 2. Barriers to employment: Language barriers 3. Facilitators and coping strategies: Language learning supports and strategies. |
| Culcasi K. 2019. | n= 45, Syrian refugee women | Jordan | Qualitative descriptive design | - Semi-structured Interviews | 1. Meaning and significance of employment: Shifting gendered performances (working and providing income), Navigation of multiple performances (having the role of men but their occupation reflecting femininity. 2. Barriers to employment: Challenges that working has created within their households (mixed emotions) 3. Facilitators and coping strategies: Seeking opportunities and positive changes that have evolved out of their coping labour. |
| Darawsheh WB, Bewernitz M, Tabbaa S, Justiss M. 2022. | Syrian Refugee  Quantitative arm  (*n* _Total_ = 254)  (*n _R_*_W_ = 159)  Qualitative arm  (9 individual interviews) | USA | Mixed methods   1. Cross-sectional survey 2. Phenomenological approach | - Quantitative arm: living difficulty scale for refugees (LDSR) - Qualitative arm:   semi structured interviews and fieldnotes | **Quantitative arm:**   1. Employment rate: NA 2. Predictors: Region in the host country,   younger age, length of time in the host country  **Qualitative arm:**  Systemic barrier – the American refugee and allocation policies, deficient role of resettlement agencies, inefficient interpretation services, and lack of support, constituted major contextual factors and shaped the experienced living and occupational difficulties by refugees.   - Occupational injustice – feelings of discrimination and social isolation and experience occupational deprivation from meaningful work activities - Interpersonal factors – age (older generation feel displaced) - Outcomes of interaction of contextual and interpersonal factors – occupational injustice, language barriers, separation from family, dependency on public benefits, limited of self-dignity limited forms of support, such as financial/economic support, can minimize opportunities for occupational participation |
| Demirci M, Kırdar MG. 2023. | Syrian refugees  Household Questionnaire:  (*n _R_*_W_ = 2,444)  Women Questionnaire  (*n _R_*_W_ = 1,995) | Turkey | Cross-sectional survey | - Governmental data records:  1. Turkey Demographic and Health Survey (TDHS) 2. The Women Questionnaire (WQ)  - From 2018 | 1. Employment rate: 6% in TDHS, 8.2% in (WQ) 2. Predictors: Region in the host country,   higher age, higher household size,  higher education, language proficiency |
| Ghorashi H. 2021. | Iranian women living in USA and Netherlands: n=20  focus group: n=8 | USA and Netherlands | Narrative inquiry approach (life stories) | - Semi-structured Interviews and focus group. | 1. Meaning and significance of employment: NA 2. Barriers to employment: Due to a strong sense of being perceived as different from others and a lack of appreciation for refugee women’s skills, abilities, and experiences, the narratives of the women in the Netherlands demonstrated little emotional connection to Dutch culture. 3. Facilitators and coping strategies: a sense of opportunity and movement among refugee women resided in the USA. |
| Grönlund A, Nordlund M. 2022. | Swedish-born  (*n* _Total_ =109,000)  Mixed ethnicity refugees  (*n* _Total_ = 11,700)  (*n _R_*_W_ = 5,598) | Sweden | Retrospective cohort | - Governmental data records: The data comes from the linked databases of STATIV and LISA. - From 1997 and 2016 | 1. Employment rate: 32% 2. Predictors: Region of origin (Middle East and North Africa lower compared to Europe),   Fertile period and parenthood were negatively related to long-term establishment (mothers at age 30), years in the host country, higher education, being married |
| Hamedanian F. 2022. | N = 30 refugee women accessing the Eurozone labour market during the COVID pandemic. | The Eurozone of the European Union. | Qualitative descriptive design | - Interviews and document analysis | 1. Meaning and significance of employment: NA 2. Barriers to employment: The racism-based gendered arrangements in the European labour market and a fragmented labour market with roots in a capitalist framework worsen the effects of the COVID pandemic on migrant women workers. Rates of unemployment indicate that female foreign nationals still face numerous challenges that may obstruct their complete integration. The economic slowdown brought on by the COVID issue affects refugee women much more than men. 3. Facilitators and coping strategies: NA |
| Huq A, Venugopal V. 2021. | n = 12 women refugee entrepreneurs | Australia | Phenomenology | - Semi-structured Interviews | 1. Meaning and significance of employment: Refugee women’s entrepreneurial journeys reveal three recurring themes: self-reconstruction, social capital, and resilience. 2. Barriers to employment: language challenges made it difficult for them to find work on the traditional job market. visa status in the nation, refugee women experience a great deal of ambiguity, losing one's previous identity, creating a new one, and embracing an unsatisfied identity. 3. Facilitators and coping strategies: NA |
| Khutso M, Frank RS, Justin RD. 2022. | n=9 International female migrant in Musina | South Africa | Case study | - Semi-structured face to face interviews. | 1. Meaning and significance of employment: NA 2. Barriers to employment: Female migrants confront a variety of obstacles that prevent them from successfully integrating into South African communities.   Facilitators and coping strategies: Female migrants in South Africa have a hard time finding employment and adopt a variety of techniques to combat hunger and unemployment, including street hawking, opening hair salons, and performing menial tasks. Female migrants have an entrepreneurial mindset since they have developed specific survival techniques. |
| Kikulwe D, Massing C, Ghadi N, Giesbrecht CJ, Halabuza D. 2021. | Syrian refugee women (n = 15) and men (n = 24) | Canada | Qualitative descriptive | - 17 focus groups with 96 newcomers | 1. Meaning and significance of employment: The credentials or skills acquired through official and informal schooling, as well as their employment position, are two interwoven facets of their life and independence during each subsequent phase of migration. 2. Barriers to employment: The participants discovered that their domestic experiences and credentials were undervalued, and that learning English was a need for employment, despite having built identities as "hard workers" in their home countries. loss of security, autonomy, credentials, and status connected to economic independence. 3. Facilitators and coping strategies: NA |
| Koyama J. 2015. | n = 98 (Refugees) | USA | Ethnography | - Semi-structured interviews and observations | 1. Meaning and significance of employment: NA 2. Barriers to employment: NA 3. Facilitators and coping strategies: Refugee women were hired by resettlement agencies worked in what was generally referred to as the "food industry" or "kitchen work". Assembling refugee women as Food Workers: Explaining the preponderance of refugee women in the food industry. |
| Manhica H, Berg L, Almquist YB, Rostila M, Hjern A. 2019. | Native Swedish  (*n* _Total_ = 3,47,255)  Intercountry  Adoptees  (*n* _Total_ = 3,47,255)  Mixed ethnicity refugees  (*n* _Total_ = 5,748)  (*n _R_*_W_ =2,678) | Sweden | Cross-sectional survey | - Governmental data records: The data comes from the linked databases of STATIV and LISA. - From 1998 and 2002 | 1. Employment rate: NA 2. Predictors: Higher education |
| Nyabvudzi T, Chinyamurindi WT. 2019. | N=20 | South Africa | Narrative inquiry | - In-depth interviews | 1. Meaning and significance of employment: In contrast to a longer-term concentration on career and professional advancement, women refugees have been more focused with short-term survival needs and acquiring essentials, the immediate need to survive. 2. Barriers to employment: Institutional limitations on employment and lived experiences (such as xenophobia, violence, and document accessibility), and problems specific to women refugees (such as sexism and work-life balance) 3. Facilitators and coping strategies: NA |
| Ortlieb R, Eggenhofer-Rehart, P, Leitner S, Hosner R, Landesmann M. 2020. | Mixed ethnicity refugees  (*n* _Total_ = 998)  (*n _R_*_W_ =324) | Australia | Cross-sectional survey | - Data from large-scale survey. - Part of a longitudinal study that started in 2016 - Cross-sectional data collected between March and May 2019 | 1. Employment rate: 24.9% 2. Predictors: Integration Year program positively helps employment for refugee women, country of origin (Iran less than Iraq), region in the country, being member in association.   Higher age, having more children.  Higher educational level, work experience in the hosting country. |
| Shaw SA, Rodgers G, Poulin P, Robinson J. 2022. | Mixed ethnicity refugees  (*n* _Total_ = 243)  (*n _R_*_W_ =124) | USA | Prospective cohort | - Four survey waves completed | 1. Employment rate: NA 2. Predictors: Region in the hosting country,   higher age, being single, extended case management, better language proficiency  No gender control |
| Senthanar S, MacEachen E, Premji S, Bigelow P. 2021. | n = 20 Syrian refugee women in Canada | Canada | Feminist grounded theory | - Semi-structured interviews with Syrian refugee women and key informants. | 1. Meaning and significance of employment: NA 2. Barriers to employment: Government Assistance Refugee (GAR) women have difficulties in their mental health and job search because they arrived with only a portion of their families and few assistances. Due to their lack of credentials and language ability, GAR women were less likely to have opportunities to demonstrate their skills in the workforce, which could have a negative impact on their mental health. 3. Facilitators and coping strategies: Privately sponsored, Blended Visa, and Refugee Claimants typically have stronger language, education, and work experience upon arrival, giving them more flexibility in terms of when and how they enter the labour market. |
| Smit R, Rugunanan P. 2014. | n = 20 Female refugees | Democratic Republic of Congo, Burundi and Zimbabwe | Qualitative descriptive | - Three focus group discussions and ten semi-structured in-depth interviews | 1. Meaning and significance of employment: NA 2. Barriers to employment: Making ends meet while seeking to secure a job, the difficulty of doing so, and the difference between involvement in the labour market then and now. Barriers to employment among refugee women’s including discriminatory practises, a lack of acknowledgment of qualifications, a language barrier, the challenge of thriving as a small business owner, "sliding down the ladder," and other factors prevent sustained revenue generating. 3. Facilitators and coping strategies: Coping strategies: using social media to 'move up' and doing part-time or sporadic jobs |
| Spehar A. 2021. | n=26 | Sweden | Qualitative descriptive | - In-depth interviews with refugee women | 1. Meaning and significance of employment: NA 2. Barriers to employment: Refugee women view the main obstacles/barriers preventing them from accessing the Swedish labour market as being a lack of institutional support during their early years in Sweden, the devaluation of ability, and difficulties with "starting over." 3. Facilitators and coping strategies: NA |
| Stempel C, Alemi Q. 2021. | Adult Afghan refugees (2006–2015 sample)  (*n* _Total_ = 4,614)  (*n _R_*_W_ =4352) | USA | Cross-sectional survey | - Governmental data records: American Community Survey (ACS) - From 2006–2015 | 1. Employment rate: 46% (*n=*2002) for the refugee between 2006 and 2015 2. Predictors: Cultural difference,   converting their higher educational credentials to economic capital, Women with physical or mental disabilities.  Facilitators: Higher levels of education, longer time in the hosting country, language proficiency |
| Thorne A. 2021. | refugee women n = 11 | Jordan | Qualitative descriptive | - In-depth interviews with refugee women | 1. Meaning and significance of employment: 2. Barriers to employment: Unable to get past the numerous obstacles that local and Syrian refugee women face while trying to find rewarding jobs. 3. Facilitators and coping strategies: Capabilities that are valued (networking, opportunities), improved, and functional (personal skills and development) impact refugee women’s employment. The skill development programme has a considerable positive impact on the participants' health and personal growth. |
| Verwiebe R, Kittel B, Dellinger F, Liebhart C, Schiestl D, Haindorfer R, et al. 2019. | n = 26 (Syria, Afghanistan, Iraq and Iran refugee women in Austria) | Austria | Qualitative descriptive | - In-depth interviews with refugee women | 1. Meaning and significance of employment: NA 2. Barriers to employment: NA 3. Facilitators and coping strategies: Opportunities seeking and seizing including personal agency factors. Using social capital: the unique contributions of volunteers, NGOs, and host families. Developing post-migration human capital: the Austrian apprenticeship system and its numerous effects on refugees' integration into the labour market. |
| Vijaya R. 2020. | Mixed ethnicity refugees  (*n* _Total_ = 12,709)  (*n* _RW_ =6,227) | USA | Cross sectional survey | - Governmental data records: American Community Survey (ACS) - From 2002–2016 | 1. Employment rate: 47.5% 2. Predictors: higher age, having young children, language proficiency, higher level of education, longer time in the hosting country. |
| Wong CK, White C, Thay B, Lassemillante ACM. 2020. | Myanmar refugees (female and males) in Australia  n = 27 | Australia | Community-based participatory research (CBPR) design | Demographic questions and four focus groups | 1. Meaning and significance of employment: Work as a significant factor affecting health. 2. Barriers to employment: NA 3. Facilitators and coping strategies: Social ties, social networks related to belonging, health and work), education and its connections to work and health. |
| Yu S-E, Kim B-Y, Jeon W-T, Jung S-H. 2012. | North Korean refugees  (*n _R_*_W_ =289) | South Korean | Cross sectional survey | Survey questions developed by the research team | 1. Employment rate: 57% 2. Predictors: Government benefits and private transfers, having young children, personal contacts (especially local) and public employment networks, governmental agencies, mass media |
