# Supplementary material for: Predictors, barriers, and facilitators to refugee women’s employment and economic inclusion: A mixed methods systematic review
Source: PLoS One. 2024 Jul 17;19(7):e0305463. doi: 10.1371/journal.pone.0305463 (PMC11253926; doi:10.1371/journal.pone.0305463)
Supplement: S2 File — (DOCX) [file pone.0305463.s002.docx]

**Critical Appraisal of the included studies**

***Supplementary File 2***

**Table 1: Critical Appraisal of Eligible Analytical Cross-Sectional Study**

| **Citation** | **Q1** | **Q2** | **Q3** | **Q4** | **Q5** | **Q6** | **Q7** | **Q8** | **Rating** |
| --- | --- | --- | --- | --- | --- | --- | --- | --- | --- |
| Baranik LE. 2021. | Y | Y | Y | Y | Y | Y | Y | Y | 100 |
| Demirci M, Kırdar MG. 2023. | Y | Y | U | Y | Y | Y | U | Y | 75 |
| Ortlieb R, Eggenhofer-Rehart, P, Leitner S, Hosner R, Landesmann M. 2020. | Y | Y | U | Y | Y | Y | U | Y | 75 |
| Stempel C, Alemi Q. 2021. | Y | Y | Y | Y | Y | Y | U | Y | 87.5 |
| Vijaya R. 2020. | Y | Y | U | Y | Y | Y | U | Y | 75 |
| Yu S-E, Kim B-Y, Jeon W-T, Jung S-H. 2012. | Y | Y | U | Y | Y | Y | U | Y | 75 |

**Y=Yes, N= No, U= Unclear, NA=Not applicable**

Q1. Were the criteria for inclusion in the sample clearly defined?

Q2. Were the study subjects and the setting described in detail?

Q3. Was the exposure measured in a valid and reliable way?

Q4. Were objective, standard criteria used for measurement of the condition?

Q5. Were confounding factors identified?

Q6. Were strategies to deal with confounding factors stated?

Q7. Were the outcomes measured in a valid and reliable way?

Q8. Was appropriate statistical analysis used?

**Table 2: Critical Appraisal of Eligible Cohort Study**

| **Citation** | **Q1** | **Q2** | **Q3** | **Q4** | **Q5** | **Q6** | **Q7** | **Q8** | **Q9** | **Q10** | **Q11** | **Rating** |
| --- | --- | --- | --- | --- | --- | --- | --- | --- | --- | --- | --- | --- |
| Arendt JN. 2022. | Y | Y | Y | Y | Y | Y | Y | Y | U | U | Y | 81 |
| Cheung SY, Phillimore J. 2017. | Y | Y | U | Y | Y | Y | U | Y | N | Y | Y | 72 |
| Grönlund A, Nordlund M. 2022. | Y | Y | U | Y | Y | Y | U | Y | U | U | Y | 63.6 |
| Manhica H, Berg L, Almquist YB, Rostila M, Hjern A. 2019. | Y | Y | U | Y | Y | Y | U | Y | N/A | N/A | Y | 63.6 |
| Shaw SA, Rodgers G, Poulin P, Robinson J. 2022. | Y | Y | N | Y | Y | Y | N | Y | Y | N/A | Y | 72.7 |

**Y=Yes, N= No, U= Unclear, NA=Not applicable**

Q1. Were the two groups similar and recruited from the same population?

Q2. Were the exposures measured similarly to assign people to both exposed and unexposed groups?

Q3. Was the exposure measured in a valid and reliable way?

Q4. Were confounding factors identified?

Q5. Were strategies to deal with confounding factors stated?

Q6. Were the groups/participants free of the outcome at the start of the study (or at the moment of exposure)?

Q7. Were the outcomes measured in a valid and reliable way?

Q8. Was the follow up time reported and sufficient to be long enough for outcomes to occur?

Q9. Was follow up complete, and if not, were the reasons to loss to follow up described and explored?

Q10. Were strategies to address incomplete follow up utilized?

Q11. Was appropriate statistical analysis used?

**Table 3: Critical Appraisal of Eligible Qualitative Research**

| **Citation** | **Q1** | **Q2** | **Q3** | **Q4** | **Q5** | **Q6** | **Q7** | **Q8** | **Q9** | **Q10** | **Rating** |
| --- | --- | --- | --- | --- | --- | --- | --- | --- | --- | --- | --- |
| Almakhamreh S, Asfour HZ, Hutchinson A. 2022. | Y | Y | Y | Y | Y | Y | N | Y | Y | Y | 90 |
| Bradley L, Bahous R, Albasha A. 2022. | Y | Y | Y | Y | Y | U | U | Y | Y | Y | 80 |
| Carlbaum S. 2022. | Y | Y | Y | Y | Y | U | U | Y | U | Y | 70 |
| Caspersz D, Casado R, Kaplanian C, Fozdar F, Baldassar L. 2022. | Y | Y | Y | Y | Y | U | U | Y | U | Y | 70 |
| Ćatibušić, B, Gallagher F, Karazi S. 2021. | Y | Y | Y | Y | Y | U | U | Y | Y | Y | 80 |
| Culcasi K. 2019. | Y | Y | Y | Y | Y | U | U | Y | U | Y | 70 |
| Ghorashi H. 2021. | Y | Y | Y | Y | Y | U | U | Y | N/A | Y | 70 |
| Hamedanian F. 2022. | Y | Y | Y | Y | Y | U | U | N | Y | Y | 70 |
| Huq A, Venugopal V. 2021. | Y | Y | Y | Y | Y | U | U | Y | Y | Y | 80 |
| Khutso M, Frank RS, Justin RD. 2022. | Y | Y | Y | Y | Y | U | U | Y | Y | Y | 80 |
| Kikulwe D, Massing C, Ghadi N, Giesbrecht CJ, Halabuza D. 2021. | Y | Y | Y | Y | Y | U | U | Y | Y | Y | 80 |
| Koyama J. 2015. | Y | Y | Y | Y | Y | U | U | Y | Y | Y | 80 |
| Nyabvudzi T, Chinyamurindi WT. 2019. | Y | Y | Y | Y | Y | U | U | Y | Y | Y | 80 |
| Senthanar S, MacEachen E, Premji S, Bigelow P. 2021. | Y | Y | Y | Y | Y | U | U | Y | Y | Y | 80 |
| Smit R, Rugunanan P. 2014. | Y | Y | Y | Y | Y | U | U | U | Y | Y | 70 |
| Spehar A. 2021. | Y | Y | Y | Y | Y | U | U | Y | Y | Y | 80 |
| Thorne A. 2021. | Y | Y | Y | Y | Y | U | U | Y | Y | Y | 80 |
| Verwiebe R, Kittel B, Dellinger F, Liebhart C, Schiestl D, Haindorfer R, et al. 2019. | Y | Y | Y | Y | Y | U | U | Y | Y | Y | 80 |
| Wong CK, White C, Thay B, Lassemillante ACM. 2020. | Y | Y | Y | Y | Y | U | U | Y | Y | Y | 80 |

**Y=Yes, N= No, U= Unclear, NA=Not applicable**

Q1. Congruity between the stated philosophical perspective and the research methodology

Q2. Congruity between the research methodology and the research question or objectives

Q3. Congruity between the research methodology and the methods used to collect data

Q4. Congruity between the research methodology and the representation and analysis of data

Q5. There is congruence between the research methodology and the interpretation of results

Q6. Locating the researcher culturally or theoretically

Q7. Influence of the researcher on the research, and vice-versa, is addressed

Q8. Representation of participants and their voices

Q9. Ethical approval by an appropriate body

Q10. Relationship of conclusions to analysis, or interpretation of the data

**Table 4: Critical Appraisal of Eligible Mixed Method Studies**

| **Citation** | **Appraisal of Quantitative Arm** | | | | | | | | **Appraisal of Qualitative Arm** | | | | | | | | | | **Rating** |
| --- | --- | --- | --- | --- | --- | --- | --- | --- | --- | --- | --- | --- | --- | --- | --- | --- | --- | --- | --- |
|  | **Q1** | **Q2** | **Q3** | **Q4** | **Q5** | **Q6** | **Q7** | **Q8** | **Q1** | **Q2** | **Q3** | **Q4** | **Q5** | **Q6** | **Q7** | **Q8** | **Q9** | **Q10** |  |
| Darawsheh WB, Bewernitz M, Tabbaa S, Justiss M. 2022. | Y | Y | Y | Y | N | N | Y | Y | Y | Y | Y | Y | Y | N | N | Y | Y | Y | 77 |

**Y=Yes, N= No, U= Unclear, NA=Not applicable**

**Appraisal of Quantitative Arm**

Q1. Were the criteria for inclusion in the sample clearly defined?

Q2. Were the study subjects and the setting described in detail?

Q3. Was the exposure measured in a valid and reliable way?

Q4. Were objective, standard criteria used for measurement of the condition?

Q5. Were confounding factors identified?

Q6. Were strategies to deal with confounding factors stated?

Q7. Were the outcomes measured in a valid and reliable way?

Q8. Was appropriate statistical analysis used?

**Appraisal of Qualitative Arm**

Q1. Congruity between the stated philosophical perspective and the research methodology

Q2. Congruity between the research methodology and the research question or objectives

Q3. Congruity between the research methodology and the methods used to collect data

Q4. Congruity between the research methodology and the representation and analysis of data

Q5. There is congruence between the research methodology and the interpretation of results

Q6. Locating the researcher culturally or theoretically

Q7. Influence of the researcher on the research, and vice-versa, is addressed

Q8. Representation of participants and their voices

Q9. Ethical approval by an appropriate body

Q10. Relationship of conclusions to analysis, or interpretation of the data
